# Editorial: Regulatory Mechanisms for Improving Cereal Seed Quality

**DOI:** 10.3389/fpls.2022.924543

**Published:** 2022-05-13

**Authors:** Vincenzo Rossi, Yingyin Yao

**Affiliations:** ^1^Council for Agricultural Research and Economics, Research Centre for Cereal and Industrial Crops, Bergamo, Italy; ^2^State Key Laboratory for Agrobiotechnology, Key Laboratory of Crop Heterosis and Utilization (MOE), Beijing Key Laboratory of Crop Genetic Improvement, China Agricultural University, Beijing, China

**Keywords:** cereals, seed, quality, nutritional value, end-use, regulatory mechanisms

In past years, enormous progress has been made in increasing the cereals yield to cope with the increase in the world population. More recently, with rising of living standard, consumers and industry have also paid great attention to quality improvement. Cereal quality is mainly determined by cereal grain and includes its processing and end-use quality, as well as the health-associated and nutritional value. Cereal quality is a complex trait affected by both genetics and environmental factors. This Research Topic aims to illustrate advances of seed quality improvement in cereals.

Maize and wheat are the most widely grown cereals (FAO, 2021) and two reviews in this Research Topic are focused on their seed quality improvement. Wu et al. wrote a comprehensive review describing the maize endosperm developmental patterns in different tissues and cell types and underlaying molecular regulatory mechanisms. The authors illustrated prospects for how knowledge of endosperm development regulation could be utilized to improve grain quality through the alteration of metabolic pathways and alteration of cellular development. Sweet corn represents one of the more familiar examples of metabolic pathways alteration, as it arises from mutations in enzymes of the starch biosynthetic pathway that impede the incorporation of glucose subunits into starch, resulting in the accumulation of free sugars in the endosperm (Lertrat and Pulam, 2007). Manipulation of basal endosperm transfer layer (BETL) development or function represents an example of cellular developmental manipulation for altering quality. Cells forming BETL are responsible for transporting metabolites from maternal tissues into the endosperm and the modulation of the relative expression levels of various classes of transporters could enhance grain filling or shift grain composition (Dai et al., 2021). This review highlighted the relevance of the gene network analyses as powerful tools to predict central regulators of gene expression modules that can be the targets of modern genetic approaches for modifying endosperm development and improving seed quality. Peng et al. provided a summary about the role of seed storage proteins (SSPs) for conferring wheat dough unique rheological properties required for the production of specific food for human nutrition. They also discussed about the efforts done for reducing specific gliadins epitopes associated with coeliac disease, while maintaining desirable end-use quality. In particular, the authors focused on the relevance of specific agricultural practices, including nitrogen and sulfur fertilization, as well as irrigation strategies, for manipulating yield and seed quality.

Due to climate change, the average global temperature is increasing continuously and is predicted to rise by 2°C until 2100, thus severely affecting crop yield and quality (Porter et al., 2014). In this Research Topic, three articles focused on the association between temperature variation and cereals quality. A slight increase in temperature induces rice chalkiness, which affects not only appearance but also milling and cooking quality (Peng et al., 2018). Wang et al. showed that application of nitrogen fertilizer during agronomical practices increased prolamin accumulation in seed, resulting in chalkiness reduction. Fan et al. grew wheat plants under field conditions to show that night-warning treatment applied at the plant vegetative stage during winter or spring significantly reduced the flag leaf senescence induced by increased warming during the post-anthesis period. Liu W. et al. used proteomics to investigate the regulatory effects of high temperature on rice grain metabolic pathways under field conditions. The results supported previous findings about the negative effects of temperature on starch and protein accumulation and composition (Tang et al., 2018). In addition, the authors identified specific molecular chaperone heat shock proteins as potential novel targets for alleviating the impact of increased warming on seed quality.

Products derived from cereals grain are relevant for human food and animal feeding, but the concentration of essential amino acids and micronutrients must reach the thresholds required for providing sufficient nutritional value. Lysine is an essential amino acid and it must be supplied through diet (Hou and Wu, 2018). The high concentration of lysine-poor prolamin storage proteins in cereals is associated with the sub-optimal nutritional quality of cereal grains. For example, barley *lys3* mutants have an increased lysine content but a reduced seed size and yield (Orman-Ligeza et al., 2020). Proteomics approaches were employed by Bose et al. to predict the impact of barley *lys3* mutations to other signaling pathways. In this study the authors provided preliminary indications to understand the nature of the pleiotropic effects associated with *ly3s* mutations, which is an essential step forward to improve lysine content without affecting yield. Even micronutrients, like Zn and Fe, are present in suboptimal levels within cereals grain and their deficiency in human diet has become one of the most common health problems (Vasconcelos et al., 2017). Tong et al. used genome-wide association studies to identify novel quantitative trait loci associated with Zn and Fe concentrations in wheat grain, thus providing essential knowledge required for improving Zn and Fe level though targeted breeding programs.

The Research Topic ends with two articles that investigate the end-use quality of cereals grain by specifically focusing on biscuits derived from the wheat flour. Liu L. et al. tested starch addition to flour of wheat cultivars with different protein content to provide information about how starch affect biscuits quality and showed that this approach can be particularly useful for improving biscuits quality using flour from wheat with low gluten content. Ma et al. focused on wheat SSPs to show that a knock-out mutation in one of the gene of the high molecular weight glutenin subunits family decreased glutenin macropolymers content, thus weakening gluten strength and improving sugar snap cookie processing quality without yield penalty.

## Author Contributions

All authors listed have made a substantial, direct, and intellectual contribution to the work and approved it for publication.

## Conflict of Interest

The authors declare that the research was conducted in the absence of any commercial or financial relationships that could be construed as a potential conflict of interest.

## Publisher's Note

All claims expressed in this article are solely those of the authors and do not necessarily represent those of their affiliated organizations, or those of the publisher, the editors and the reviewers. Any product that may be evaluated in this article, or claim that may be made by its manufacturer, is not guaranteed or endorsed by the publisher.
